# Comprehensive Network-Based Analyses Reveal Novel Renal Function-Related Targets in Acute Kidney Injury

**DOI:** 10.3389/fgene.2022.907145

**Published:** 2022-07-04

**Authors:** Yang Zhang, Jieru Cai, Wei Lu, Sujuan Xu, Mengdi Qu, Shuan Zhao, Xiaoqiang Ding

**Affiliations:** ^1^ Department of Nephrology, Zhongshan Hospital, Fudan University, Shanghai, China; ^2^ Shanghai Medical Center of Kidney Disease, Shanghai, China; ^3^ Kidney and Dialysis Institute of Shanghai, Shanghai, China; ^4^ Kidney and Blood Purification Key Laboratory of Shanghai, Shanghai, China; ^5^ Department of Anesthesiology, Zhongshan Hospital, Fudan University, Shanghai, China

**Keywords:** acute kidney injury, weighted gene co-expression network analysis, renal function, targeted therapeutics, transcriptome

## Abstract

**Background:** Acute kidney injury (AKI) is a common clinical syndrome with limited methods of treatment and diagnosis. Although several molecules associated with AKI have been discovered, molecular mechanisms underlying AKI still remain unclear. Weighted gene co-expression network analysis (WGCNA) is a novel method to uncover the relationship between co-expression genes and clinical traits at the system level.

**Methods:** First, by employing WGCNA in transcriptional data on 30 patients with well/poor functioning kidney graft, we identified two co-expression modules that were significantly related to serum creatinine (SCr). Second, based on the modules, potential small molecular compound candidates for developing targeted therapeutics were obtained by connectivity map analysis. Furthermore, multiple validations of expression in space/time were carried out with two classical AKI models *in vivo* and other five databases of over 152 samples.

**Results:** Two of the 14 modules were found to be closely correlated with SCr. Function enrichment analysis illustrated that one module was enriched in the immune system, while the other was in the metabolic process. Six key renal function-related genes (RFRGs) were finally obtained. Such genes performed well in cisplatin-induced or cecal ligation and puncture-induced AKI mouse models.

**Conclusion:** The analysis suggests that WGCNA is a proper method to connect clinical traits with genome data to find novel targets in AKI. The kidney tissue with worse renal function tended to develop a “high immune but low metabolic activity” expression pattern. Also, ACSM2A, GLYAT, CORO1A, DPEP1, ALDH7A1, and EPHX2 are potential targets of molecular diagnosis and treatment in AKI.

## Introduction

AKI is a complicated syndrome involving rapid loss of kidney function, defined by decreasing urine output, increasing serum creatinine, or both (Ronco et al., 2019). Causes of AKI are traditionally classified by anatomical features, namely prerenal, intrarenal, and postrenal causes (Cerdá et al., 2008; Chawla et al., 2017), or by particular pathophysiological processes, including cardiorenal (Zannad and Rossignol, 2018), nephrotoxic (Uber and Sutherland, 2020), hepatorenal (Ginès et al., 2018), and sepsis-associated AKI (Poston and Koyner, 2019). Due to its complex etiology and pathophysiology, further research is needed on the molecular mechanisms of AKI.

Serum creatinine (SCr) is the most used biomarker for assessing AKI (Bellomo et al., 2004) and is involved in defining and staging AKI (Thomas et al., 2015). Still, it appears to be inaccurate and insensitive in the early identification and evaluation of AKI. Only a significant drop in GFR or substantial parenchymal injury that occurs can lead to a serum creatinine increase (Mehta et al., 2007; Wen and Parikh, 2021), and the production of creatinine is closely related to patients’ age, gender, and muscle volume. Due to traditional biomarkers’ limitations on assessing AKI, diverse novel biomarkers need to be discovered based on specific pathophysiology processes. For example, kidney injury molecule-1 (KIM1) is a transmembrane glycoprotein expressed by proximal tubular cells, which was correlated with the severity of tubular injury (Vaidya et al., 2010; Yang et al., 2015). However, there is still a long way to go for clinical adoption, and an ideal biomarker for precise recognition and staging is needed.

Meanwhile, due to the complexity of etiology, the treatment of AKI has had little progress recently. Monitoring fluid, electrolyte, and acid-base balance, early recognition of complications, and kidney replacement therapy are the limited methods we can carry out (Bagshaw and Wald, 2017; Meraz-Muñoz et al., 2021; Bouchard and Mehta, 2022). The drugs targeting the pathophysiological process of kidney injury are finite and need further study.

Nowadays, genomic studies provide new techniques and perspectives for studying novel biomarkers of early kidney injury, bringing people’s awareness of more accurate and efficient molecular diagnostic and therapeutic methods (Liu et al., 2019). In consideration of biomarkers’ diagnostic and therapeutic role, an investigation combined with clinical features is optimal. The Limma/DESeq2/EdgeR algorithm to identify differentially expressed genes (DEGs) is the common method for discovering novel targets in AKI and other diseases. However, due to analyzing the transcriptome data singly, they lack clinical information, and only two groups (treatment vs. control) can be analyzed. On the contrary, WGCNA can combine the clinical data (even the continuous variable such as serum creatinine), and multiple groups can be taken into analysis. Therefore, WGCNA is a proper method for discovering renal function-related genes. Moreover, WGCNA is a systematic strategy for conjoining network analysis and clinical manifestation, containing five classical steps: 1) construction of a gene co-expression network, 2) identification of modules of genes with similar expression patterns, 3) relating modules to external information, 4) construction of module relationship, and 5) finding the key drivers in interesting modules. They can identify novel biomarkers and therapeutic targets with a pivotal character (Langfelder and Horvath, 2008). Therefore, WGCNA provides a strategy for connecting genes with certain biological features. It is widely applied for identifying tumor biomarkers. Chen et al. (2019) identified that acetyl-CoA acetyltransferase and metabolism-related pathways are vital for the progression of clear cell renal cell carcinoma. WGCNA was also used to identify hub genes and describe transcriptome characteristics in other kidney diseases. Shen et al. (2021) indicated that five hub genes play vital roles in the pathogenesis of lupus nephritis for their capacities to be a diagnostic and therapeutic target. Beckerman et al. (2017) conducted a 95-sample transcriptome data analysis by WGCNA, suggesting a regulatory role of fibrosis in connecting kidney function and gene expression. In contrast, rare studies adopt WGCNA in AKI research due to the lack of biopsy samples in AKI patients.

However, a single analysis or single database can cause false-positive results. We adopted not only WGCNA but also differentially expressed genes (DEGs) and space-time dual validation in six databases with more than 182 samples in human/rat/mouse models. In the present study, gene expression profiles from GEO datasets were adopted and analyzed by WGCNA, and a gene co-expression network was constructed. Subsequently, we obtained the key RFRGs as diagnostic and therapeutic targets filtrated through multiple datasets and analysis and then validated *in vivo*. Our research provides a molecular landscape correlated with clinical traits of renal function.

## Materials and Methods

### Data Collection

**FIGURE 1:**
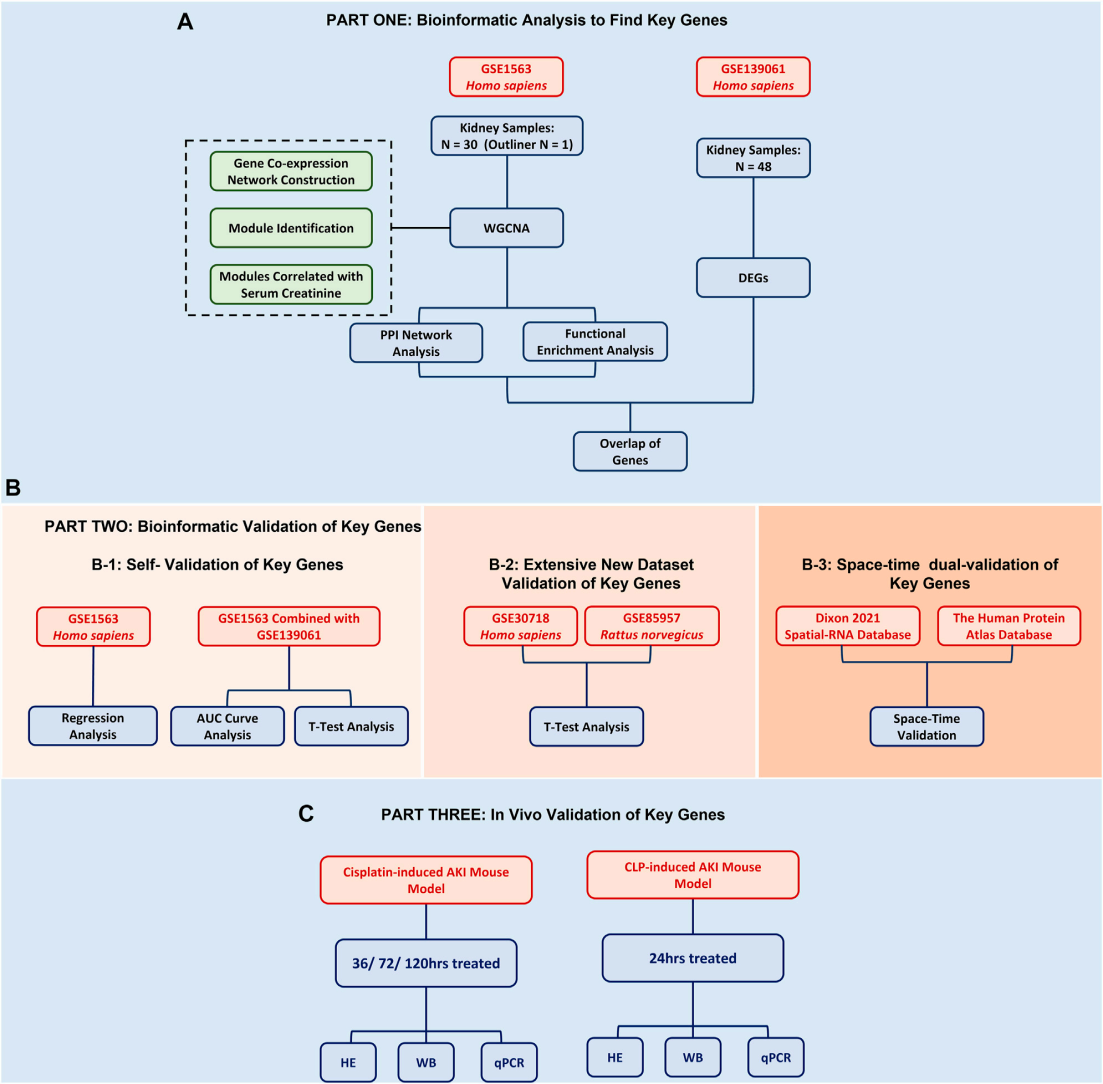
Flowchart of the bioinformatic analysis and experimental validation.

In this study, in order to find renal function-related genes (RFRGs), 30 samples of the GSE1563 data set were used for WGCNA analysis, and 48 samples of the GSE139061 data set were used for DEG analysis. For further validation, 47 samples of GSE30718 and 57 samples of GSE85957 were analyzed, respectively. The aforementioned data set was obtained from the GEO database (https://www.ncbi.nlm.nih.gov/geo/, 20/03/2022). For potential therapeutics, connectivity map analysis was adopted through the L1000 database (https://clue.io/, 20/03/2022). For space-time dual validation of RFRGs, transcriptional data from GTEx (https://gtexportal.org/home/, 20/03/2022), immunohistochemical data from The Human Protein Atlas Database (https://www.proteinatlas.org/, 20/03/2022), and scRNA-Seq data from Kidney Interactive Transcriptomics (http://humphreyslab.com/SingleCell/, 20/03/2022) were achieved and analyzed (Figure 1).

### Co-expression Network Construction and Functional Analysis

The WGCNA R package (version: 1.70–3) was performed to construct a scale-free network. The soft-thresholding power was set as 10, according to the scale-free topology criterion with a correlation coefficient of 0.9. Highly correlated genes were divided into gene modules using cluster analysis, but the module was excluded if the size was less than 30. The cut height threshold was set as 0.9 to explore more gene modules. The gray module represented background genes that belonged to none of the modules.

### Identification of Differentially Expressed Genes

Limma (linear models for microarray data, DOI: 10.1093/nar/gkv007) is a differential expression screening method based on generalized linear models; here, we used the R package limma (version 3.40.6) for differential analysis to obtain differentially expressed genes between the AKI group and the REF group (control) based on GSE139061.

### Functional Enrichment Analysis

For gene ontology (GO) annotation and Kyoto encyclopedia of genes and genomes (KEGG) pathway enrichment analysis, we employed the R package clusterProfiler (version 3.14.3) to obtain the results. The minimum gene was set as 5, and the maximum gene was set as 5,000. An FDR value of <0.01 was considered statistically significant.

### Protein–Protein Interaction Network Construction

Topological overlap matrix was achieved through WGCNA and was transferred to the network into the edge and node list files Cytoscape can read. Subsequently, the network diagrams were plotted by Cytoscape 3.9.0.

### Mouse AKI Model

Eight-week-old male C57BL/6J mice (weighing 20–25 g) obtained from Shanghai Jihui Laboratory Animal Care Co. LTD., Shanghai, China, were housed under appropriate conditions. For the establishment of the cisplatin-induced AKI model, mice in the cisplatin group were intraperitoneally injected with a single dose of cisplatin (Sigma-Aldrich) at 20 mg/kg, while mice in the control group received saline only. Kidneys were obtained for analysis after 36 and 72 h, respectively. For the establishment of the sepsis-induced AKI model, the cecum was perforated three times using a 20-gauge needle. In sham animals, the cecum was exteriorized without puncture. Kidneys were obtained for analysis after 24 h. One part of renal tissues was then fixed with 4% paraformaldehyde, embedded in paraffin wax, and sliced for hematoxylin-eosin (H&E) staining. The other part was stored under −80°C for protein or mRNA extraction. All the protocols were performed in accordance with the National Institutes of Health Guide for the Care and Use of Laboratory Animals and were approved by the Animal Care and Use Committee of Zhongshan Hospital. All the experiments were replicated at least twice.

### Western Blotting

Mice kidneys were lysed in TRI Reagent (Sigma-Aldrich), according to the manufacturer’s guidelines. Samples were then assessed by 10% SDS-PAGE and transferred to polyvinylidene fluoride membranes (IPVH00010, Millipore). After blocking with 5% milk, the membranes were incubated with primary antibodies overnight at 4°C against the following proteins: ACSM2A (1:1,000, A15563, Abclonal), EPHX2 (1:1,000, A1885, Abclonal), ALDH7A1 (1:1,000, A8629, Abclonal), GLYAT (1:1,000, A14100, Abclonal), CORO1A (1:1,000, A9300, Abclonal), DPEP1 (1:1,000, A6289, Abclonal), and ACTIN (1:2,000, GTX110003, GeneTex). After incubation with secondary antibodies (1:2,000, Jackson ImmunoResearch Inc.), the bands of the target proteins were finally visualized by the LAS-3000 detection system.

### RNA Isolation and Real-Time RT-PCR

Total RNA from mice kidney tissues was isolated using the TRI Reagent (Sigma-Aldrich). PrimeScript RT Master Mix and SYBR Premix ExTaq™ (TaKaRa) on QuantStudio 5 (Thermo Fisher Scientific) were used to carry out the reverse transcription and real-time RT-PCR, respectively. ACTIN was used as internal control, and the 2^−ΔΔCt^ method was applied to calculate the fold change differences of the experimental groups compared to the control group. The following primers (Sangon Biotech (Shanghai) Co., Ltd.) 5’ to 3’ were used (*Mus musculus*):

**Table:** 

ACSM2A	F	TGG​GGG​AAT​GAG​ATT​TCC​TGC
	R	CTT​CAC​TCA​GTT​CTC​GGA​AGC
EPHX2	F	ACC​ACT​CAT​GGA​TGA​AAG​CTA​CA
	R	TCA​GGT​AGA​TTG​GCT​CCA​CAG
ALDH7A1	F	CTC​TGC​TGA​TCC​ATC​ATC​CCC
	R	CCC​CAG​CTT​CCA​TTA​TAC​ACG
GLYAT	F	GGT​TTA​TGG​GAC​CGT​CTA​TCA​CA
	R	GTG​GGA​CTG​GGA​ACT​TTG​AAT​C
CORO1A	F	AGG​CAA​GAC​TGG​ACG​AGT​AGA
	R	CCA​TCC​GGG​ATC​TCC​CAC​A
DPEP1	F	GCA​CAA​CGA​CTT​GCC​TTG​G
	R	ATG​CGG​TGT​ATC​ACA​TCC​ATC
KIM1	F	AGG​CGC​TGT​GGA​TTC​TTA​TG
	R	AAG​CAG​AAG​ATG​GGC​ATT​GC
ACTIN	F	GGC​TGT​ATT​CCC​CTC​CAT​CG
	R	CCA​GTT​GGT​AAC​AAT​GCC​ATG​T

### Statistical Analysis

The pROC R package (version 1.18.0) was used to plot the ROC curve and compute the corresponding area under the curve (AUC). The bar graph data were presented as mean ± standard deviation (SD). Welch’s *t*-test and unpaired *t*-test were used to compare data between the two groups. Simple linear regression was used to analyze data between corresponding gene expression and clinical characteristics. The experiments were performed with at least three biological replicates. The statistical analyses were performed in GraphPad Prism (version 9.3.0), and *p*-values less than 0.05 were considered statistically significant.

## Results

### Co-expression Network Construction for AKI in the Kidney Transplant Database

**FIGURE 2:**
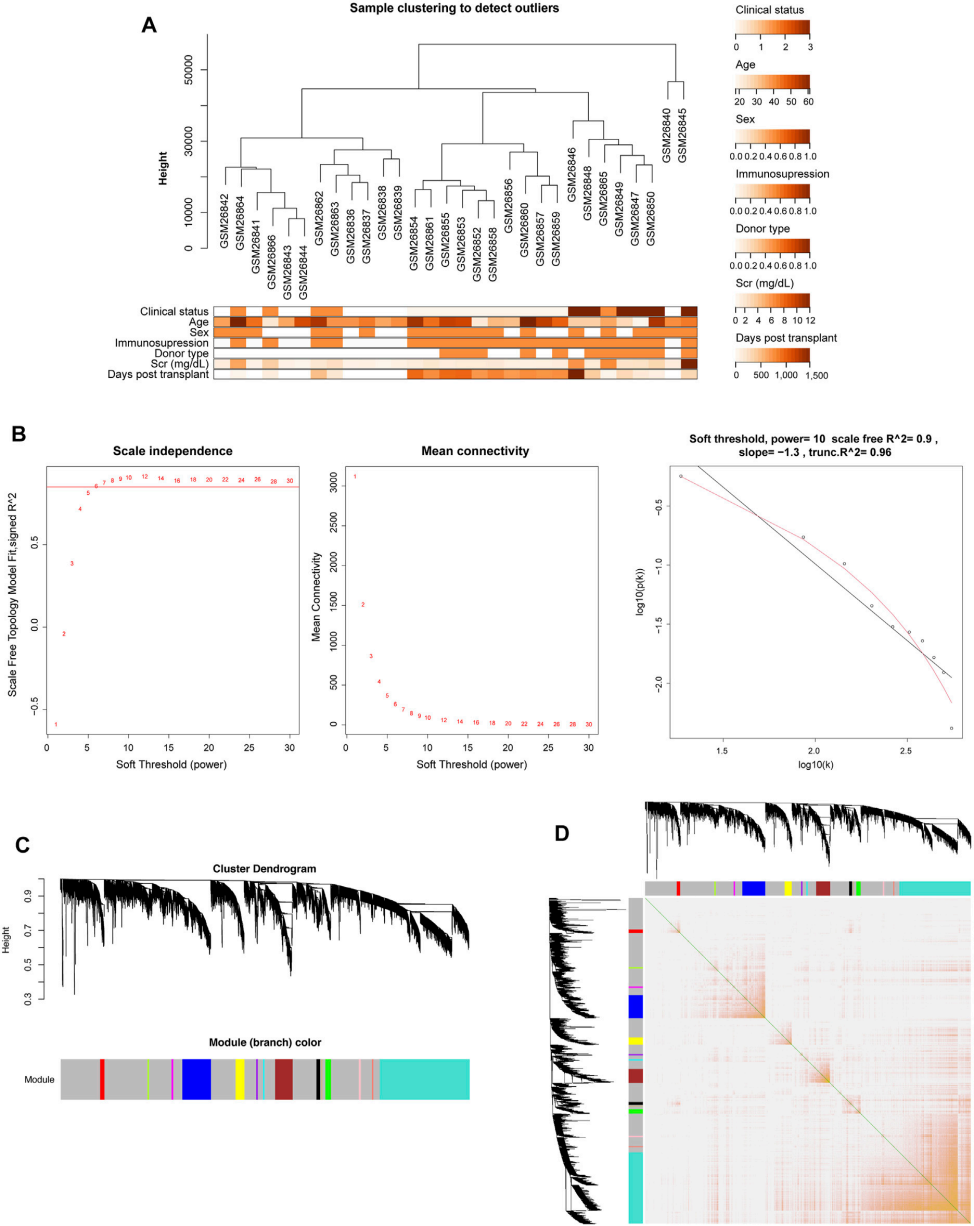
Co-expression network construction of GSE1563 analyzed by WGCNA. **(A)** Hierarchical clustering dendrogram of kidney samples in GSE1563 and the corresponding clinical trait heatmap. **(B)** Analysis of appropriate soft-thresholding powers. The red line indicates a correlation coefficient of 0.85. The second plot shows average network connectivity under weighting coefficients. The third plot indicates the correlation of log (k) and log [P (k)]. **(C)** Clustering dendrograms of 9,155 genes in 30 samples, from which 14 co-expression modules were constructed with different colors. **(D)** Heatmap plot depicts the TOM among all genes in the analysis, which shows the interactions among co-expression modules. The stronger intensity of orange indicates greater overlaps.

The gene expression data and corresponding clinical information of GSE1563 were obtained from the GEO database, including four groups: healthy normal donor kidneys (n = 9; clinical status: 0), well-functioning transplants with no clinical evidence of rejection (n = 10; clinical status: 1), transplants with renal dysfunction without rejection (n = 5; clinical status: 2), and kidneys undergoing acute rejection (n = 7; clinical status: 3). The genes that were not available (NA) and duplicate values of the same gene symbol except the maximum one were removed. After clustering analysis, there was one outlier sample (Supplementary Figure S1) (Flechner et al., 2004). Therefore, 30 samples and 9,155 genes were finally obtained. Figure 2A depicts the cluster tree of the 30 samples and the corresponding clinical information.

Due to the scale-free characteristics of the co-expression network, the soft thresholding power was considered carefully as 10 with a proper correlation coefficient >0.9 (Figures 2B,C). After the weighting coefficient was determined, the gene expression profiles of 9,155 genes could be transformed into the adjacency matrix, TOM, and dissTOM. Subsequently, 14 co-expression modules except the gray one were constructed (Figure 2C and Supplementary Material S2). Moreover, dissTOM with hierarchical clustering showed that the main modules were independent of each other in the network (Figure 2D).

### Identification of Renal Function-Related Modules in the Co-expression Network

**FIGURE 3:**
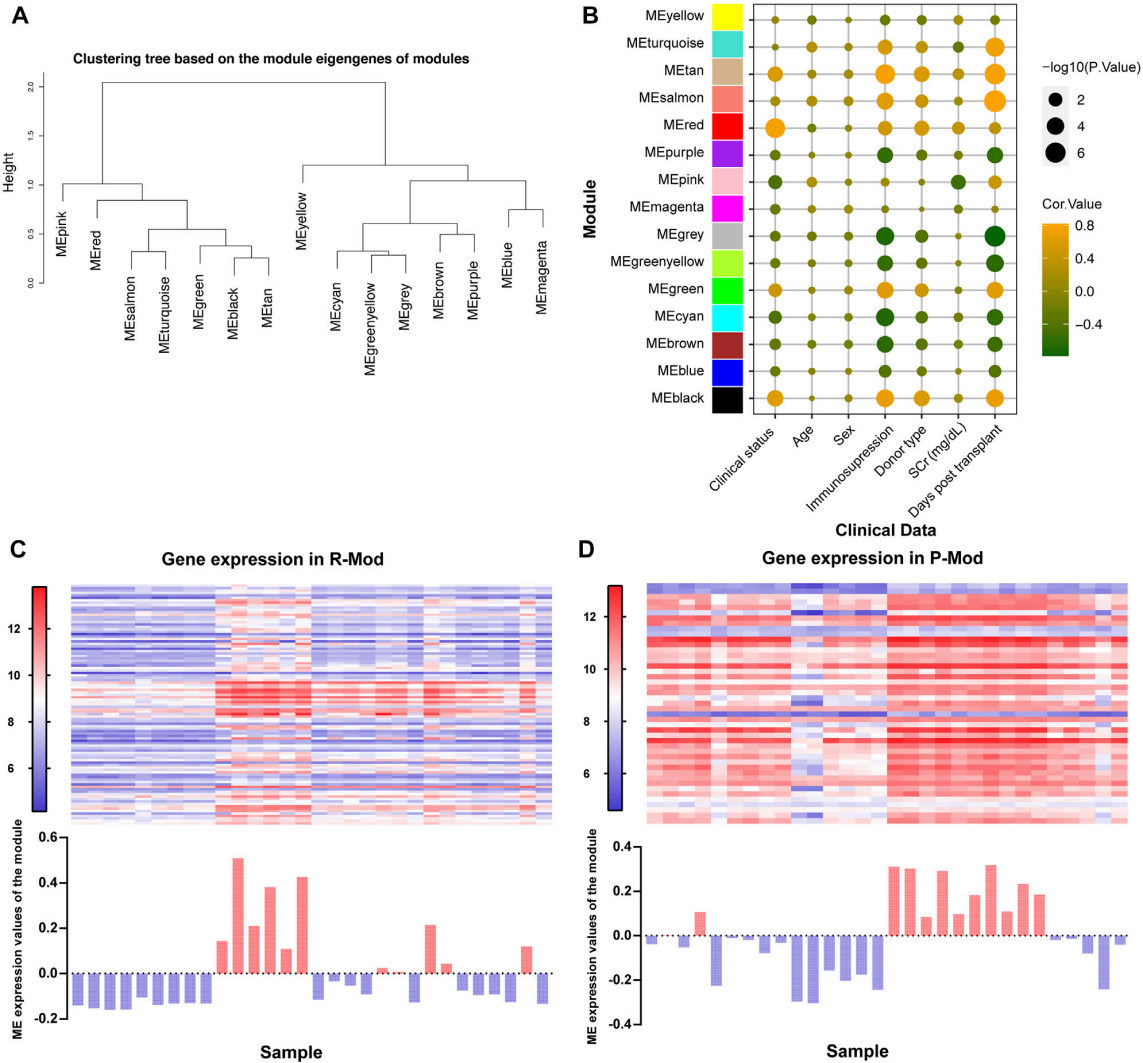
Relationships between modules and clinical traits based on GSE1563. **(A)** Hierarchical clustering dendrogram of MEs. **(B)** Bubble plot of the association between the ME and clinical traits. The color represents the correlation coefficient, while the size shows the corresponding -log (*p*-value). **(C,D)** Upper heatmap shows the expression levels of genes P-/R-Mod in each sample. The lower histogram shows the corresponding ME expression values of P-/R-Mod in each sample.

Module eigengenes (ME) are the first principal component in each module, which are obtained through WGCNA and represents the overall expression level of the corresponding module (Long et al., 2021). We conducted the clustering tree based on ME and found that pink and red modules (P-Mod and R-Mod) were in the same major branch but different minor branches (Figure 3A). Furthermore, the Pearson correlation coefficients for the MEs and the clinical information of each module were calculated to identify which module was related to the specific clinical traits. The P-Mod (R = −0.52, *p* = 0.003) and R-Mod (R = 0.39, *p* = 0.03) were significantly associated with serum creatinine, a vital and common clinical index representing the renal function (Figure 3B and Supplementary Material S3). Interestingly, the P-Mod was only related to the clinical status and SCr and independent of other clinical traits, which indicated that it could be a more specific renal function-related gene set. Then, we analyzed the gene expression and MEs in P- and R-Mod. It was obvious that there were two opposite expression patterns in the two modules, which was consistent with the previously mentioned R-value (Figures 3C,D).

### Biological Characteristics and Potential Targeted Therapeutics of Renal Function–Related Modules

**FIGURE 4:**
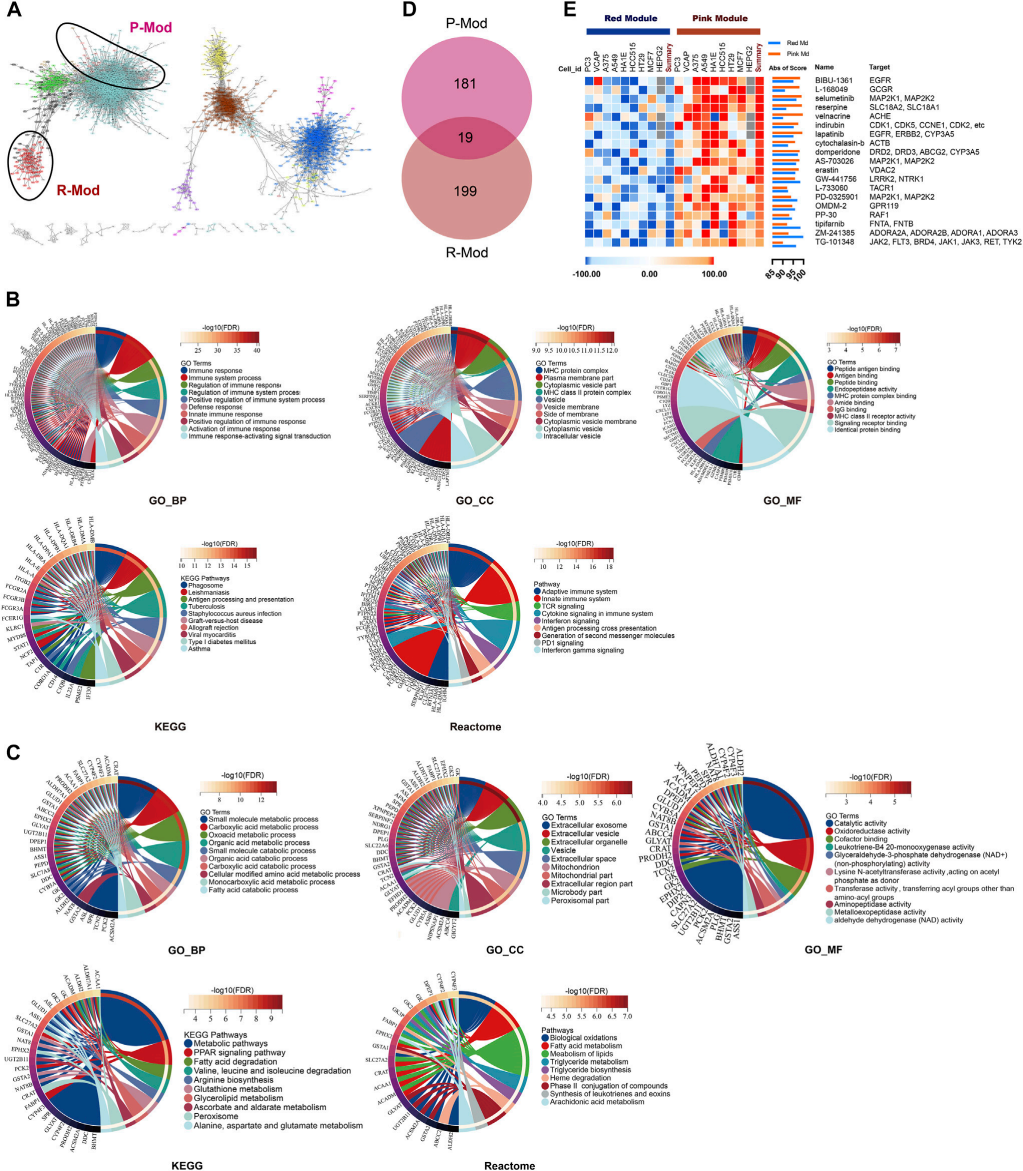
PPI network, functional enrichment analysis, and potential targeted therapeutics based on SCr-related modules. **(A)** Interaction network analysis based on WGCNA. Each dot is color-coded by its corresponding module. R-/P-Mod are marked with circles. **(B,C)** Chord plots of the enriched GO/KEGG/Reactome terms of R- **(B)**/P-Mod **(C)**. **(D)** Venn plot depicts the overlap of 19 small molecular compounds identified in both red-module-targeted (Score ≥90) and pink-module-targeted (Score ≥90) compounds. **(E)** Heatmap of expression pattern scores of 19 targeted compounds in P- and R-Mod, respectively.

The genes in the same module are highly co-expressed and might share potential biological significance. We transferred the dissTOM to the network into edge and node list files Cytoscape could read, and the PPI map showed the same as the ME clustering tree (Figure 4A). The P- and R-Mod were in one major “continent,” but there was a long distance between each, which might suggest totally different biological processes related to renal function during AKI. Thus, in order to explore the specific biological functions, we performed GO, KEGG, and Reactome functional enrichment analyses. The enriched functions of the R-Mod focused on “immune response,” “MHC protein complex,” “peptide antigen binding,” “antigen processing and presentation,” and “adaptive immune system,” while the enriched P-Mod focused on “small-molecule metabolic process,” “extracellular exosome,” “catalytic activity,” “metabolic pathways,” and “biological oxidation” (Figures 4B,C and Supplementary Material S4). Combined with the WGCNA result, the kidney tissue with worse renal function tended to develop a “high immune but low metabolic activity” expression pattern.

Therefore, through comparison of the expression pattern of R- and P-Mod with the pattern of small molecular compounds by connectivity map analysis (Subramanian et al., 2017), 218 and 199 targeted compounds were discovered, respectively. Nineteen of the compounds were identified as potential efficient therapeutics in both groups (Figures 4D,E).

### Identification of Key Renal Function-Related Genes

**FIGURE 5:**
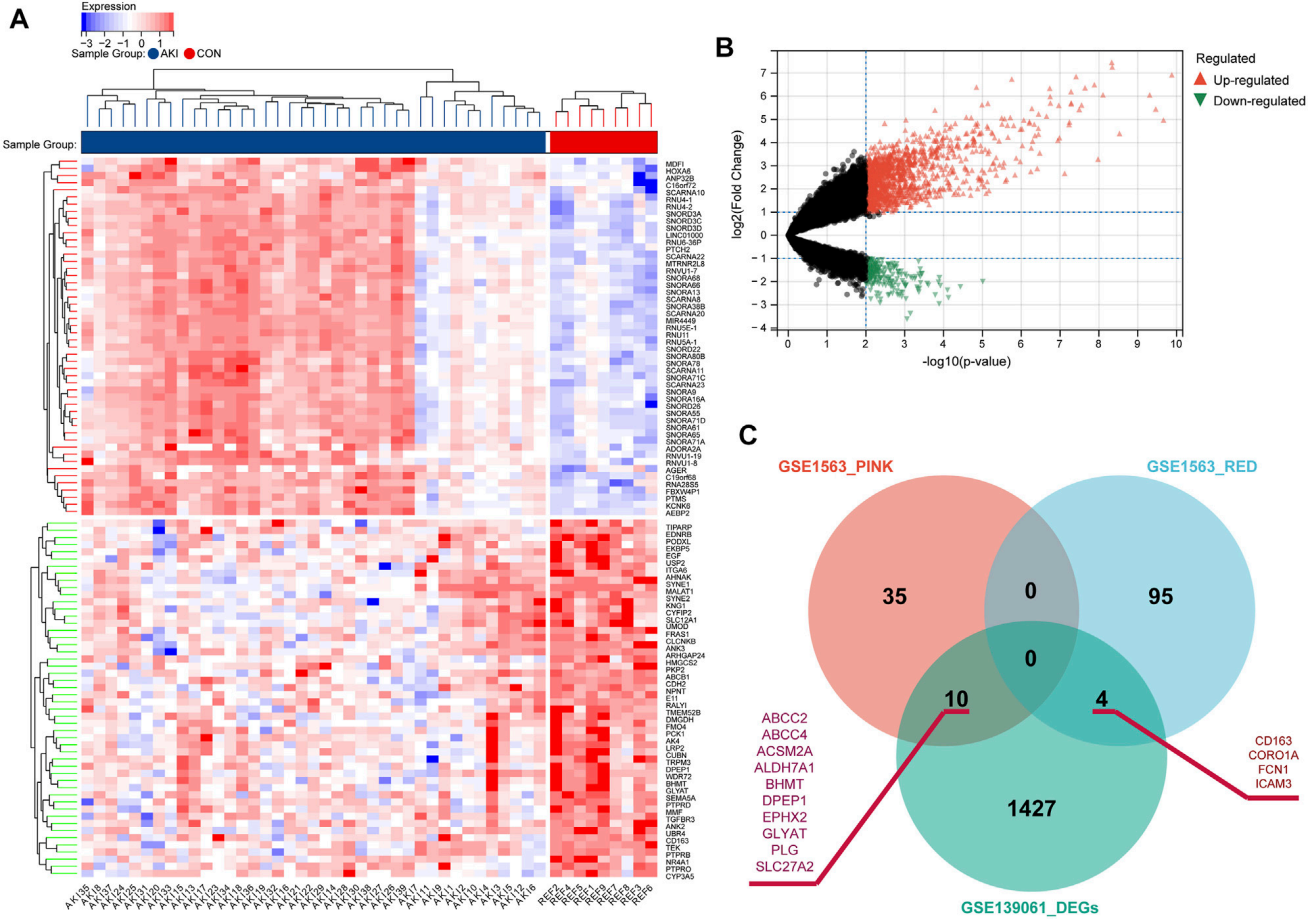
Identification of the differentially expressed genes in AKI patients based on GSE139061 and screening of key genes. **(A)** Heatmap of top 50 upregulated and 50 downregulated differentially expressed genes from AKI and REF groups. **(B)** Volcano plot of the DEGs. **(C)** Venn plot depicts the overlap of 14 genes identified in both DEGs from GSE139061 and genes in SCr-related modules from GSE1563, which are defined as RFRGs (renal function-related genes).

In order to explore the key RFRGs in such two modules and extend the universality, DEG analysis was performed between control samples (n = 9) and AKI samples (n = 39) from GSE139061 (Figures 5A,B). At the threshold of *p*-value < 0.01 and log_2_|FC| of >1, 1,441, DEGs were screened out. Subsequently, 10 genes (*ABCC2*, *ABCC4*, *ACSM2A*, *ALDH7A1*, *BHMT*, *DPEP1*, *EPHX2*, *GLYAT*, *PLG*, and *SLC27A2*) were obtained from both the P-Mod and the DEGs, and four genes (*CD163*, *CORO1A*, *FCN1*, and *ICAM3*) were obtained from both the R-Mod and the DEGs, which were considered as renal function-related genes (RFRGs) (Figure 5C).

### Validation of Key Renal Function-Related Genes

**FIGURE 6:**
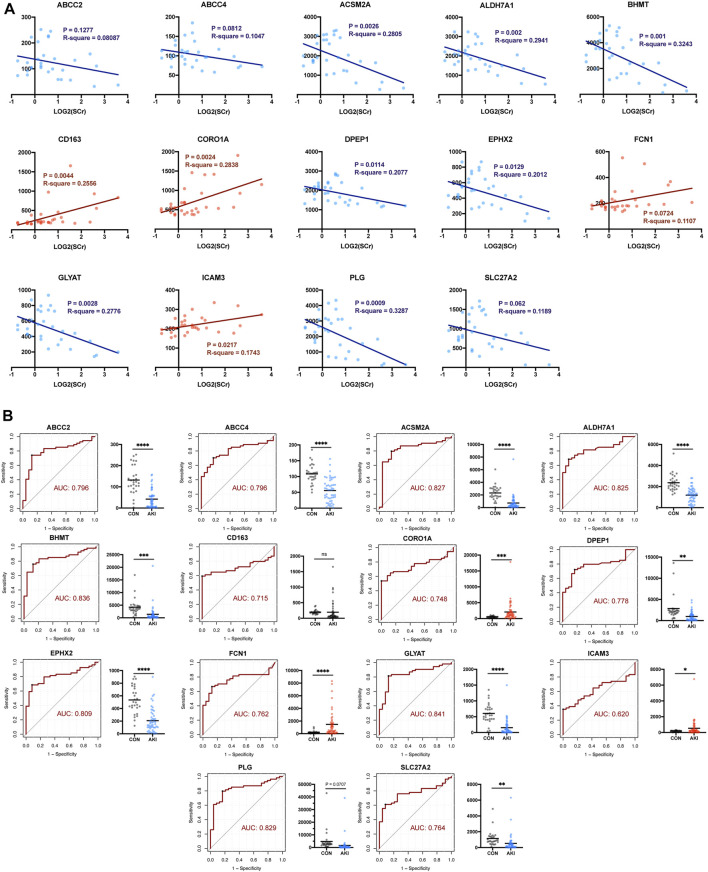
Self-validation of RFRGs by regression analysis based on GSE1563 and GSE139061. **(A)** Regression analysis between the value of log_2_(SCr) and transcriptional level of RFRGs. **(B)** ROC curve and AUC value are calculated, and expression levels between the control and AKI group are shown in the histogram plot. *p < 0.05, **p < 0.01, ***p < 0.001, and ****p < 0.0001 vs. the control group.

Given that there might be false-positive results, further validation in GSE1563 was carried out by simple linear regression analysis between log_2_(SCr) and each of the 14 RFRGs. The relationship between log_2_(SCr) and ABCC2 (*p* = 0.1277), FCN1 (*p* = 0.0724), and SLC27A2 (*p* = 0.062) was absent of enough statistical significance (Figure 6A). Moreover, after normalization of the median of the normal renal function group in GSE1563 and GSE139061, the receiver operating characteristic (ROC) of each of 14 RFRGs with a total of 78 samples verified that all RFRGs showed adequate specificity and sensitivity to AKI, and only CD163 performed poorly in the Welch’s *t*-test between control and AKI groups (Figure 6B).

**FIGURE 7:**
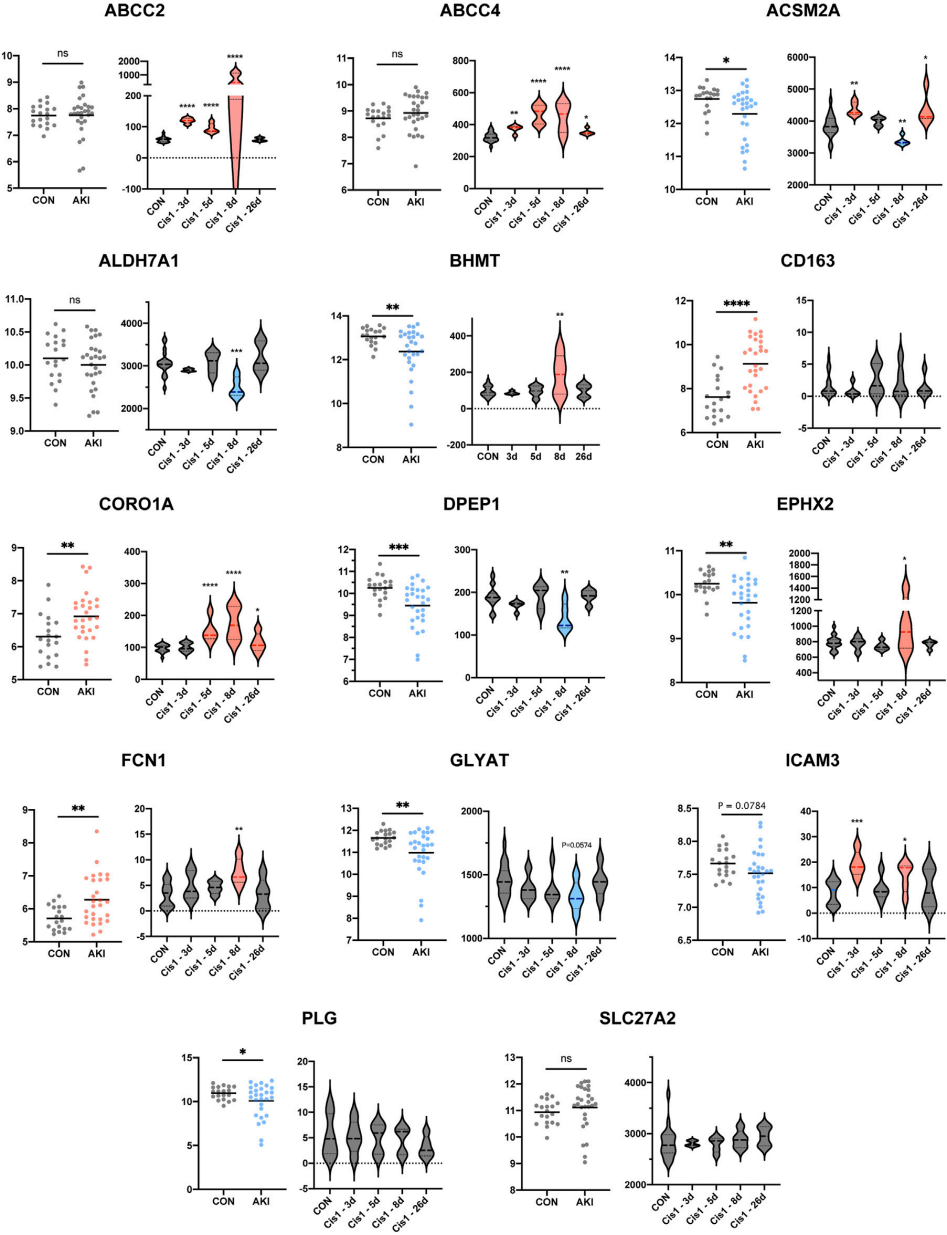
Extensive new dataset validation of key genes based on GSE30718 and GSE85957. Histogram plot shows expression level between control and AKI groups based on human datasets GSE30718 (left). Different expressions along with time course are depicted in boxplots (right). *p < 0.05, **p < 0.01, ***p < 0.001, and ****p < 0.0001 vs. the control group.

Since the aforementioned validation was limited to discovery sets (GSE1563 and GSE139061) themselves, the human AKI dataset (GSE30718) and rat AKI dataset with time-course data (GSE85957) were both added to the following extensive validation (Famulski et al., 2012; Pavkovic et al., 2014). As a result, there were more negative (ALDH7A1, PLG, CD163, and SLC27A2) or contradictory results (EPHX2, ICAM3, ABCC2, and ABCC4) and also some consistent findings (ACSM2A, GLYAT, CORO1A, DPEP1, and FCN1). The genes (ACSM2A, GLYAT, CORO1A, DPEP1, and FCN1) with a similar trend to the aforementioned results were more possible to be the vital target in AKI (Figure 7). In order to narrow the range and uncover the functions of RFRGs, we scored the genes after different parts of validation, which we would summarize in the following scoring (Figure 9A).

### Space-Time Dual Analysis of Key Renal Function-Related Genes

**FIGURE 8:**
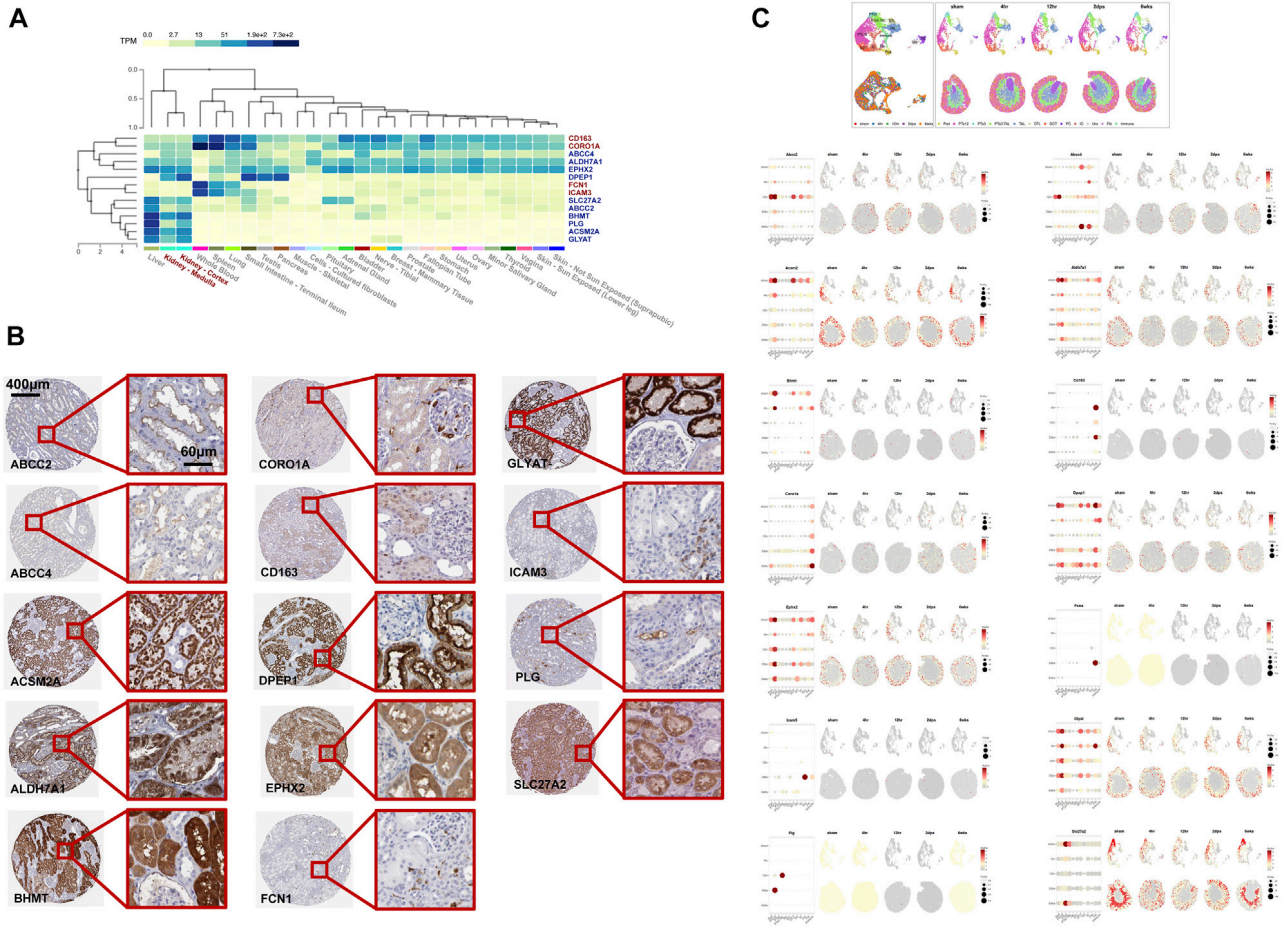
Space-validation of RFRGs based on The Human Protein Atlas Database and space-time dual-validation of RFRGs based on the Dixon 2021 scRNA-Seq database. **(A)** Transcriptional distribution of RFRGs in different organs based on datasets in the GTEx database. **(B)** Immunohistochemical staining of RFRGs depicted specific translational distribution in renal tissue based on The Human Protein Atlas Database. **(C)** UMAPs of cell type clustering and spatial plots of increased resolution for major cell types. Spatial expression of RFRGs along the female injury time course. Pod, podocytes. PTs12, proximal tubule segments 1–2. PTs3, proximal tubule segments 3. TAL, thick ascending limb. DTL, descending thin limp of loop of Henle. DCT, distal convoluted tubule. PC, principal cells. IC, intercalated cells. Uro, urothelium. Fib, fibroblasts.

In order to locate the RFRGs, we first analyzed the transcriptional data from the GTEx database to find the organ location of such genes (Figure 8A) (Dixon et al., 2022; The Genotype, 2013). Ten RFRGs from P-Mod were expressed highly in the kidney, which was downregulated in AKI. On the contrary, four RFRGs from R-Mod were expressed highly in blood but lowly in the kidney, which was upregulated in AKI. Consistent with the function enrichment analysis, exuberant metabolic activity in the kidney shut down, while immune-related cells migrated from the blood to the kidney as renal function declined and AKI deteriorated. To confirm the specific cell location of such genes in the kidney, immunohistochemical data from the human protein atlas database were obtained (Figure 8B; Table 1) (Sjöstedt et al., 2020). Overall, RFRGs in P-Mod were mainly expressed in the tubules, especially in proximal tubules. As proximal tubules are the most vulnerable part of the kidney during AKI, the RFRGs in P-Mod might contribute to the conversion of metabolic activity in proximal tubules, leading to their apoptosis/necrosis/proliferation. Understandably, RFRGs in R-Mod like CORO1A, FCN1, and ICAM3 had weak staining, which was mainly expressed in the circulation system. The upregulation of the RFRGs in R-Mod might play a key role in the invasion of immune cells and their proinflammatory functions.

**TABLE 1:** Staining distribution of 14 RFRGs.

	Location	
	Glomeruli	Tubules
ABCC2	—	Proximal tubules (microvilli) +++
ABCC4	—	+
ACSM2A	—	+++
ALDH7A1	—	Collecting ducts +; Distal tubules +; Proximal tubules (cell body) +++
BHMT	—	Distal tubules ++; Proximal tubules (cell body) +++
CD163	—	++
CORO1A	—	+
DPEP1	—	Proximal tubules (cell body) ++; Proximal tubules (microvilli) +++
EPHX2	—	++
FCN1	—	—
GLYAT	—	+++
ICAM3	—	+
PLG	—	Proximal tubules (microvilli) ++
SLC27A2	—	Proximal tubules (cell body) +++

Space-time dual analysis was then carried out in RFRGs using a spatial transcriptomics database (Figure 8C) (Wu et al., 2018; Kirita et al., 2020). Most RFRGs in P-Mod were located in proximal tubules and were downregulated as AKI processed (4 h s, 12 h s) but raised as AKI recovered (2dps, 6wks), except ABCC2 and ABCC4. All RFRGs in R-Mod were located in fibroblasts or immune cells and were upregulated in AKI vs. sham group. The results also supported the aforementioned hypothesis.

### Validation of Transcriptional and Translational Expressions in Cisplatin-Induced and Sepsis-Induced AKI Models *In Vivo*

**FIGURE 9:**
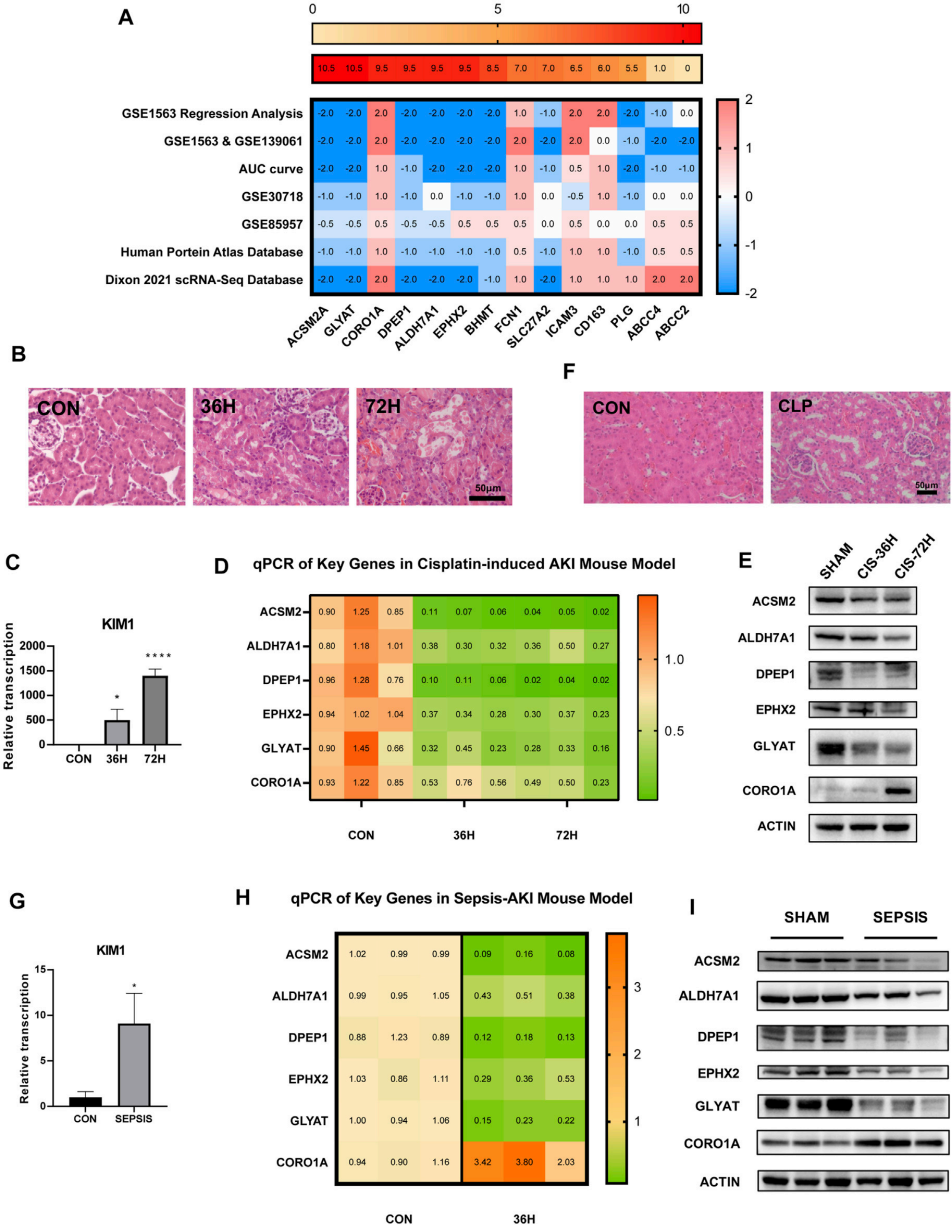
Validation of the mRNA and protein expression of RFRGs. **(A)** Score of the 14 RFRGs. The aforementioned heatmap shows the total scores of each gene, and the heatmap below shows the exact scores of seven analyses. **(B,C)** Degree of renal injury was measured by H&E staining and relative mRNA levels of KIM1 of kidney sections from the cisplatin-induced AKI model at different time points. **(D,E)** Relative mRNA level and representative immunoblot analysis of the top six RFRGs in the cisplatin-induced AKI model at different time points. **(F,G)** Degree of renal injury was measured by H&E staining and relative mRNA levels of KIM1 of kidney sections from the CLP-induced AKI model at different time points. **(H,I)** Relative mRNA level and representative immunoblot analysis of the top six RFRGs in the CLP-induced AKI model at different time points. *p < 0.05, **p < 0.01, ***p < 0.001, and ****p < 0.0001 vs. control group.

Based on all the aforementioned analyses, we scored each of the 14 RFRGs. According to different biological significance of each dataset and analysis, different weights of each part were identified (see details in Table 2). The top six genes with scores >9 (*ACSM2A*, *GLYAT*, *CORO1A*, *DPEP1*, *ALDH7A1*, and *EPHX2*) were finally selected to be confirmed further in the AKI model *in vivo* (Figure 9A).

**TABLE 2:** Standard of scoring of 14 RFRGs.

Weight (Total Points)	GSE1563 Regression Analysis	GSE1563 & GSE139061	AUC Curve		GSE30718	GSE85957
	±2	±2	±2		±1	±0.5
If negative correlation/declining trend and *p* < 0.05	−2	−2	−2		−1	−0.5
If negative correlation/declining trend and *p* < 0.1	−1	−1	−1		−0.5	0
If positive correlation/increasing trend and *p* < 0.05	2	2	2		1	0.5
If positive correlation/increasing trend and *p* < 0.1	1	1	1		0.5	0
If *p* > 0.1	0	0	0		0	0
	HPA database					
**Weight (total points)**	**±1**					

If negative correlation/declining trend in GSE1563 Regression analysis/GSE1563 & GSE139061	Stained strongly in tubules	−1
	Stained weakly in tubules	−0.5
If positive correlation/increasing trend in GSE1563 Regression analysis/GSE1563 & GSE139061	Stained in tubules	1
	No staining in tubules	0.5
	**Dixon 2021 ** **scRNA-** **Seq** ** Database**	
**Weight (total points)**	**±2**	
If declining trend vs. control	Max expression was high	−2
	Max expression was low	−1
If increasing trend vs. control	Max expression was high	2
	Max expression was low	1

ACSM2, ALDH7A1, DPEP1, EPHX2, and GLYAT showed both lower transcriptional and translational levels in not only the cisplatin-induced AKI model but also the cecum ligation and puncture (CLP)-induced AKI model (Figures 9B–I). Interestingly, the mRNA expression declined in the cisplatin-induced AKI model (Figure 9D), but the protein expression of CORO1A increased in both models consistent with the aforementioned analysis.

Taken together, ACSM2, ALDH7A1, DPEP1, and GLYAT performed well not only in every part of bioinformatic exploration and validation but also in experiments *in vivo*. Probably, due to heterogeneity of rat/mice model or the doses of cisplatin, EPHX2 and CORO1A showed contradictory changes in GS85957 or the mRNA level in the cisplatin induced-AKI model, respectively.

## Discussion

To better diagnose and treat AKI precisely, it is imperative to investigate molecular targets with high specificity and sensitivity. By using multiple analysis strategies, our study identified biomarkers related to clinical features, which emphasizes the connection between clinical information and gene expression differences, and these biomarkers could be valuable therapeutic targets. We then validated these hypothetical genes in several series of samples, and further investigation *in vivo* showed a similar tendency.

We mainly chose the kidney transplant patients’ samples for analysis as microarray analysis of human kidneys with AKI has a limited account for such kidney being seldomly biopsied. However, kidney transplants’ samples could be excellent resources, because all kidney transplants experience AKI, and they are extensively followed up with regular clinical tests and kidney punctures (Famulski et al., 2012). In this study, two sample series were first involved for recognizing key genes. Thirty kidney samples connected to kidney transplant rejection that concluded the SCr level were analyzed by using the WGCNA analysis strategy, and 48 human renal biopsy samples from patients with AKI were taken into DEG analysis strategy. WGCNA is a network analysis method based on coefficients, which evaluates the correlation patterns between microarray samples (Langfelder and Horvath, 2008; Kakati et al., 2019). During the WGCNA, a gene co-expression network is obtained for identifying clusters of highly interconnected genes as different modules. Aimed to find interesting modules, modules are then related to external information including clinical features, resulting in a group of genes closely connected to the external data. The optimal sample size of this model is higher than 15, and when the sample size is more than 20, the effect is better, and the results are more reliable (Langfelder and Horvath, 2008). We used 30 kidney samples in WGCNA and recognized 14 modules, and further analysis indicated two modules (P-, R-Mod) were closely coupled with elevated SCr levels in AKI patients. Function enrichment analysis of P- and R-Mod showed genes in the R-Mod are linked to various immune functions, while genes in the P-Mod are mainly associated with metabolic pathways; 144 genes are recognized in WGCNA. Another sample series adopted DEG analysis because of lack of clinical features, and 1,441 genes were taken up. Overlap of those two groups revealed 14 genes closely related to both sample series. Furthermore, we validated these 14 genes in several sample series by different methods. The time and space distribution of genes was also inspected based on the Human Protein Atlas database and Dixon 2021 scRNA-Seq database. We examined the predictive effect of these genes on AKI, and the score was assigned according to the results of each test. The top six genes were ACSM2A, GLYAT, CORO1A, DPEP1, ALDH7A1, and EPHX2.

A similar trend has been observed in the cisplatin- and CLP-induced AKI models. A decrease in ACSM2A, GLYAT, DPEP1, ALDH7A1, and EPHX2 had been observed at transcription and expression levels, which is consistent with our hypothesis. However, it is opposed to our expectations that the mRNA level of CORO1A did not increase during the injury in the cisplatin-induced AKI model. Other than the heterogeneity of the mouse model and dose of cisplatin, the main effect of CORO1A is the immune function, and the influence of tissue irrigation in sampling cannot be ignored, which may reduce the detection of immune cells. There are several studies consistent with our results. Current research confirmed that ACSM2A paralleled the function and maturation of proximal tubular cells, which is verified in several animal models and patients’ samples (Watanabe et al., 2020). A genome-wide association study on chronic kidney disease also identified the relationship between ACSM2A and eGFR (Ledo et al., 2015; van der Sluis, 2018), showing its potential of being a kidney injury biomarker. Genome-wide association analyses revealed DPEP1 as a kidney disease risk gene in kidney proximal tubules, and further molecular investigation on cisplatin-associated kidney injury indicated its regulative function in ferroptosis and apoptosis (Nakagawa et al., 1992; Guan et al., 2021). EPHX2, encoding soluble epoxide hydrolase (sEH), is widely investigated in various renal injuries and transplantation (Lee et al., 2008; Liu et al., 2012; Mota-Zamorano et al., 2019; Duflot et al., 2021). One of the important functions of sEH is metabolizing epoxyeicosatrienoic acids (EETs), which is an anti-inflammatory substant. Based on the protective properties of EETs, inhibition of sHE is a potential target for kidney protection (Manhiani et al., 2009; Greite et al., 2020; Lin et al., 2020; He et al., 2021). However, the exact functions of six RFRGs in the kidney still need further study.

There are still several limitations to this research. First, although multiple analysis methods and models from different AKI causes validated the six genes, further investigations on a specific role in molecular mechanisms of these genes in AKI still need to be performed. Second, due to the sample source of GSE1563, a transplant kidney dataset, the potential AKI-targeted compounds, and function enrichment analysis need further validation not only in a bioinformatic way but also in *in vitro* and *in vivo* experiments. Therefore, more profound explorations are needed to uncover the molecular landscape of AKI.

## Conclusion

In summary, P- and R-Mods enriched in immune activity and metabolism were identified to be associated with renal function in AKI by WGCNA. Based on two modules, 19 potential targeted small molecular compounds were identified. After validation with multiple analyses of five datasets, ACSM2A, GLYAT, CORO1A, DPEP1, ALDH7A1, and EPHX2 were considered as the key RFRGs and were confirmed at both mRNA and protein levels in two classic AKI models *in vivo*. In this study, we explored novel molecular targets and drug candidates, which may provide new insights for diagnosis and treatment for AKI.

## Data Availability

The original contributions presented in the study are included in the article/Supplementary Material; further inquiries can be directed to the corresponding authors.

## Abbreviations

AKI, acute kidney injury; WGCNA, weighted gene co-expression network analysis; RFRGs, renal function-related genes; SCr, serum creatinine; DEG, differentially expressed gene; ME, module eigengenes; CLP, cecum ligation and puncture.
