# Supplementary figures and images for: Comprehensive Network-Based Analyses Reveal Novel Renal Function-Related Targets in Acute Kidney Injury
Source: Front Genet. 2022 Jul 4;13:907145. doi: 10.3389/fgene.2022.907145 (PMC9289212; doi:10.3389/fgene.2022.907145)

Supplementary Figure 1. Sample clustering to detect outliers.

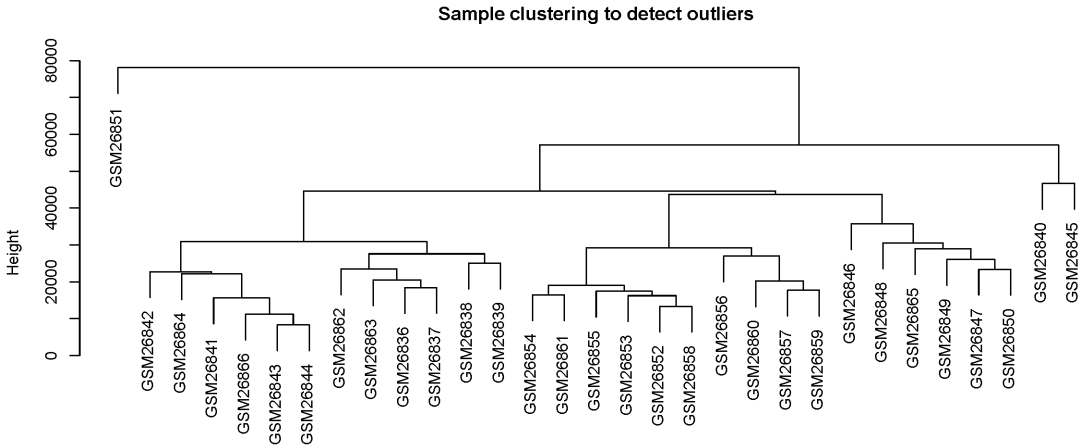

Supplement: Supplementary file 3 [file DataSheet1.PDF]
